# Investigation of the Influence of Process Parameters on the Physicochemical and Functional Properties of Oil-Based Composites

**DOI:** 10.3390/ma18153447

**Published:** 2025-07-23

**Authors:** Anita Zawadzka, Magda Kijania-Kontak

**Affiliations:** 1 Department of Engineering and Chemical Technology, Cracow University of Technology, 24 Warszawska St., 31-155 Cracow, Poland; 2 Department of Civil Engineering, Cracow University of Technology, 24 Warszawska St., 31-155 Cracow, Poland; magda.kijania-kontak@pk.edu.pl

**Keywords:** composite, oil block, compression strength, absorbability, waste cooking oil, building materials, cleaner production

## Abstract

The increasing consumption of edible oils has resulted in a parallel rise in waste cooking oil (WCO), a harmful waste stream but one that also represents a promising raw material. In this study, oil-based binders were synthesised from WCO using various reagents: Sulfuric(VI) acid, hydrobromic acid, acetic acid, salicylic acid, glycolic acid, zinc acetate, ethanol, hydrogen peroxide, and their selected mixtures. The manufacturing process was optimised, and the composites were evaluated for physicochemical and mechanical properties, including compressive strength, bending strength, and water absorption. The best performance was observed for composites catalysed with a mixture of sulfuric(VI) acid and 20% hydrogen peroxide, cured at 240 °C, yielding compressive and bending strengths of 5.20 MPa and 1.34 MPa, respectively. Under modified curing conditions, a compressive strength of 5.70 MPa and a bending strength of 0.75 MPa were obtained. The composite modified with glycolic acid showed the lowest water absorption (3%). These findings demonstrate how catalyst type and curing parameters influence composite structure, porosity, and mechanical behaviour. The study provides new insights into the process–structure–property relationships in oil-based materials and supports the development of environmentally friendly composites from waste feedstocks.

## 1. Introduction

Contemporary engineering technologies are in constant search of new materials that combine high mechanical strength with strict environmental compliance. Traditional feedstocks used in composite manufacture—such as synthetic resins and polymer-based materials—are typically derived from petrochemical sources, which raises their carbon footprint and produces waste that is difficult to manage. Conventional construction materials, by contrast, are cement-based, which results in significant CO_2_ emissions—primarily from the calcination of calcium carbonate and the high energy demand of the production process [1,2]. Therefore, it is essential to develop new, more sustainable solutions that couple high mechanical strength with environmentally friendly production methods [3,4].

One innovative approach involves using waste cooking oil (WCO) as a feedstock for composite production. WCO-based composites are drawing increasing interest from researchers and industry alike, as their manufacture aligns with the principles of the circular economy [5]. In recent years, the growing consumption of edible oils has driven a parallel rise in the amount of WCO generated worldwide—both in households and, especially, in the food-service industry. Global vegetable-oil production exceeded 220 million metric tons in the 2023/24 season and is projected to reach roughly 239 million metric tons by 2028 [6,7]. Because WCO is unfit for consumption and is harmful to both human health and the environment, it constitutes a problematic waste stream that demands proper disposal [6,8]. Its physicochemical properties depend on numerous factors, including the degree of prior use, thermal conditions, type of contaminants, and water content [9]. The frying process in vegetable oils can exceed 190 °C, triggering a series of chemical reactions [10]. The original properties of the oils deteriorate significantly during the frying stage, as triglycerides undergo both thermal and chemical degradation. Oxidative and hydrolytic reactions can occur, producing free fatty acids (FFA), glycerol, monoglycerides, and diglycerides, and increasing the proportion of saturated and monounsaturated fatty acids [11,12,13].

To mitigate the adverse effects of improper disposal, WCO can be transformed into value-added products such as biofuel, bioplasticizer, biolubricant, bio-asphalt, 3D-printing resin, and polymers [14,15,16,17]. Recently, researchers have been focusing on novel materials that incorporate a binder derived from modified waste cooking oil together with an aggregate [14,18,19,20]. The incorporation of waste cooking oil into composites not only cuts down on hard-to-degrade waste streams but also lowers the demand for virgin raw materials, contributing to resource efficiency [21].

In recent years, there has been a dynamic increase in research on composite materials produced from waste oils, including waste cooking oil. Staroń provided a detailed overview of the processes and properties of WCO-based composites used in construction, analysing parameters such as curing conditions and water absorption [19].

Studies comparing different types of waste oils indicate that the composition of the raw material significantly influences the crosslinking process and structural parameters of the materials. For instance, research from 2024 demonstrated that natural additives such as turmeric or thymol can enhance not only the functional properties (e.g., odour reduction) but also the mechanical performance of oil-based composites [20,22]. The study emphasises WCO’s potential to replace petroleum-based polymer precursors in sustainable material development. This review outlines chemical pathways for converting WCO into various polymers—such as polyurethanes, epoxies, polyhydroxyalkanoates, acrylics, and alkyd resins—through processes involving ester groups and double bonds. It also summarises the catalysts, initiators, and crosslinkers used in these transformations [14]. The paper [23] emphasises the importance of optimising processing conditions in hardening processes for material stability and carbon footprint.

The resulting WCO-based composites display favourable mechanical properties, notably tensile and compressive strength. Moreover, their hydrophobic character and resistance to chemical attack open up uses across diverse sectors—from construction to the production of biodegradable packaging [24,25]. Their low cost and the ability to produce them from waste feedstocks make WCO-based composites an attractive alternative to conventional synthetic counterparts.

The manufacturing process comprises several key steps: homogenising the WCO with an acidic catalyst; enriching the mixture with aggregate and, where required, additives that confer specific performance attributes; and finally heat-curing the material to achieve solidification [19]. Optimising the oil-composite manufacturing process makes it possible to tailor the materials’ mechanical strength, heavy-metal leachability, water absorption, and application-specific properties (including hydrophobicity and oleophobicity) [26,27,28,29,30]. To broaden the application spectrum of WCO-based composites, natural additives are incorporated into the formulations. Composites have already been produced that are enriched with, among others, thymol, turmeric, cobylac, hops, or salicylic acid [20,22,31]. To date, the literature has concentrated on producing composites whose binder is modified with sulfuric(VI) acid—a logical choice given its low cost and high efficiency. However, environmental concerns and the acid’s corrosive effect on processing equipment are driving the search for alternative substances capable of yielding equally durable oil composites. Studies exploring the manufacture of oil composites with other reagents remain scarce, revealing a research gap that must be addressed by developing WCO-based composites with improved physicochemical properties and by broadening the palette of catalysts assessed for WCO-composite production.

Despite increasing interest in the valorisation of waste cooking oil (WCO), there remains a noticeable scientific deficit in the systematic investigation of key manufacturing parameters for oil-based composites. Most studies focus on proof-of-concept approaches or environmental aspects, while critical factors such as curing conditions, catalyst selection, and catalyst-to-oil ratio are often insufficiently explored or standardised. This study aims to address this gap by evaluating the effect of various catalytic systems and processing parameters on the mechanical and physicochemical properties of WCO-based composites. The results contribute to a deeper understanding of process–structure–property relationships and provide a foundation for further optimisation and scalability of this sustainable material system.

This study explored the feasibility of producing composites whose binder is waste cooking oil (WCO). The WCO was modified with sulfuric(VI) acid, another inorganic acid (hydrobromic acid), a carboxylic acid (acetic acid), an aromatic hydroxy-carboxylic acid (salicylic acid), an α-hydroxy acid (glycolic acid), the zinc salt of acetic acid (zinc acetate), ethanol, hydrogen peroxide, and various mixtures of these reagents. By systematically comparing different catalytic systems and curing parameters, this work contributes to the advancement of sustainable material technologies. The findings are relevant for industries seeking eco-friendly and resource-efficient alternatives to traditional composites.

## 2. Methodology

### 2.1. Oil-Based Binder

The oil binders were prepared by mixing WCO with the chemical reagents (catalysts) in the quantities specified in Table 1. The WCO was collected from a restaurant serving traditional Polish cuisine and then filtered to remove food residues. The catalyst-to-catalysed-WCO mass ratio (cat./WCOcat) was set at 0.05, 0.10, or 0.15. Where two catalysts were used, the first reagent was blended with the WCO and, after 3 min of homogenisation, the second was added. The mixture was then kept on a mechanical stirrer for a further 5 min, transferred to aluminium moulds and heat-cured in accordance with the experimental schedule (Table 1).

### 2.2. Preparation of Oil Composites

**Figure 1:**
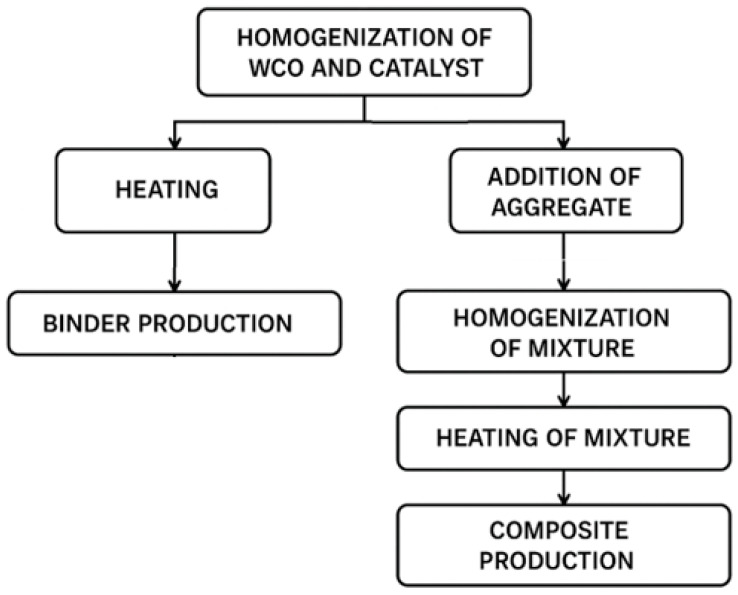
The stages of production of oil binders and WCO-based composites.

The oil composites were produced in the same manner as the oil binders (Section 2.1), except that sand was added at the final stage and the mixture was homogenised for an additional 5 min. The proportion of catalysed oil was kept constant at 22.5% relative to the mass of sand (a fraction of 0.5–1.4 mm, in a dry-air state). The resulting paste was transferred to aluminium moulds and heat-cured. The process parameters were identical to those that had yielded solidified oil binders. The stages of production of oil binders and WCO-based composites are shown in Figure 1. In Table 2, the parameter sets that produced monolithic (solid) materials are highlighted in grey.

### 2.3. Physicochemical Characterisation of the Compositesoil-Based Binder

The oil composites were first tested for compressive strength on a Zwick/Roell Z100 universal tester (initial load 25 N; cross-head speed 1 kN min^−1^), with the force applied to the top face of each specimen. Samples that withstood compressive stresses greater than 3.185 MPa (i.e., a failure load above 1 kN) were then evaluated for bending strength. Bending tests were carried out in accordance with [32] and bending with [33]. Prismatic oil-composite specimens were subjected to three-point bending under hydraulic loading until failure, and the breaking load was recorded for each sample. To evaluate water absorption, composite specimens of approximately 20 g each were immersed in distilled water for 72 h at a composite-to-water mass ratio of 1:15. After immersion, the pH of every solution was measured, the samples were removed, surface moisture was blotted off, and the specimens were weighed. Surface morphology was examined with an Apreo 2 S LoVac scanning electron microscope (Thermo Fisher Scientific, Waltham, MA, USA). The molecular structure of the composites was analysed by Fourier-transform infrared spectroscopy (FT-IR) using a Nicolet iS5 spectrometer (Thermo Scientific, Waltham, MA, USA) over the 500–4000 cm^−1^ range.

Approximation profiles were created to determine the values of the independent parameters to obtain the most favourable estimated values of the output factor. The approximated output factor values for combinations of input factor values were converted to a utility scale. The approximation profile approach has the potential to reduce the need for extensive experimental testing. Utility values of the dependent variable can range from 0.0 (undesirable) to 1.0 (highly desirable). In addition, Pareto charts were created from the analysis of variance to graphically represent the effect of independent variables on dependent variables (standardised effects). The vertical line shown in the graphs corresponds to the assumed level of significance (α = 0.05) and separates statistically significant from non-significant effects. Statistical analysis was carried out in version 13.3 of STATISTICA by StatSoft^®,^ Tulsa, OK, USA.

## 3. Results and Discussion

### 3.1. Formation of the Oil Binder

**Figure 2:**
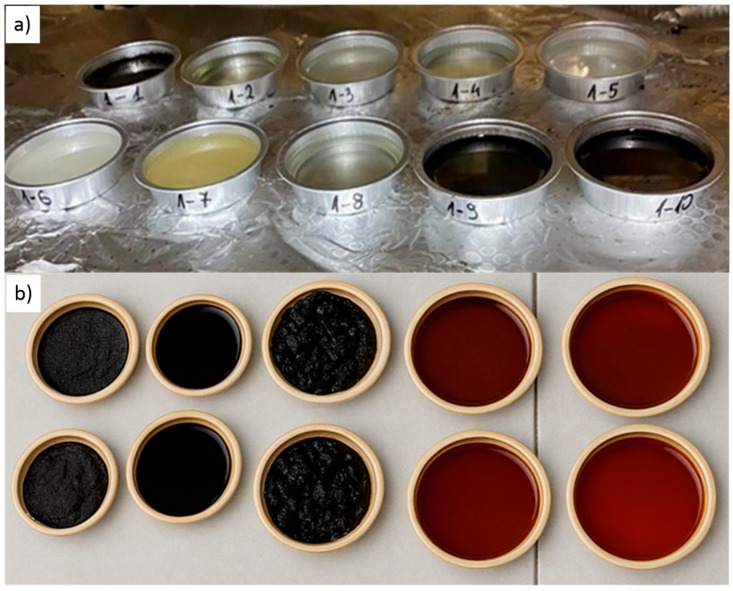
Representative mixtures of WCO and catalysts: (**a**) before curing; 1-1 (with sulfuric(VI) acid); 1-2 (with acetic acid), 1-3 (with salicylic acid); 1-4 (with glycolic acid); 1-5 (with zinc acetate); 1-6 (with hydrogen peroxide); 1-7 (with hydrobromic acid); 1-8 (with ethyl alcohol); 1-9 (with 10% hydrogen peroxide + acetic acid); 1-10 (with 20% hydrogen peroxide + acetic acid); (**b**) after curing (at 180 °C for 12 h, Cat./WCOcat 0.15).

The colour of the WCO–catalyst mixtures varied with the catalyst employed: it ranged from colourless/transparent (acetic acid, salicylic acid, glycolic acid, zinc acetate), through yellow (hydrobromic acid), to dark brown (sulfuric(VI) acid containing hydrogen peroxide) (Figure 2a). After heat curing, every oil binder became noticeably darker, from dark brown to brown-black (Figure 2b). Specimens cured at the higher temperature (240 °C) exhibited the darkest colour, likely due to sintering or increased polymerization at the elevated temperature.

Throughout this work, sample codes are composed as follows: the numeral denotes the processing parameters listed in Table 2, while the letters indicate the catalyst type as given in Table 1.

### 3.2. Composition Analysis of the Oil Binders

**Figure 3:**
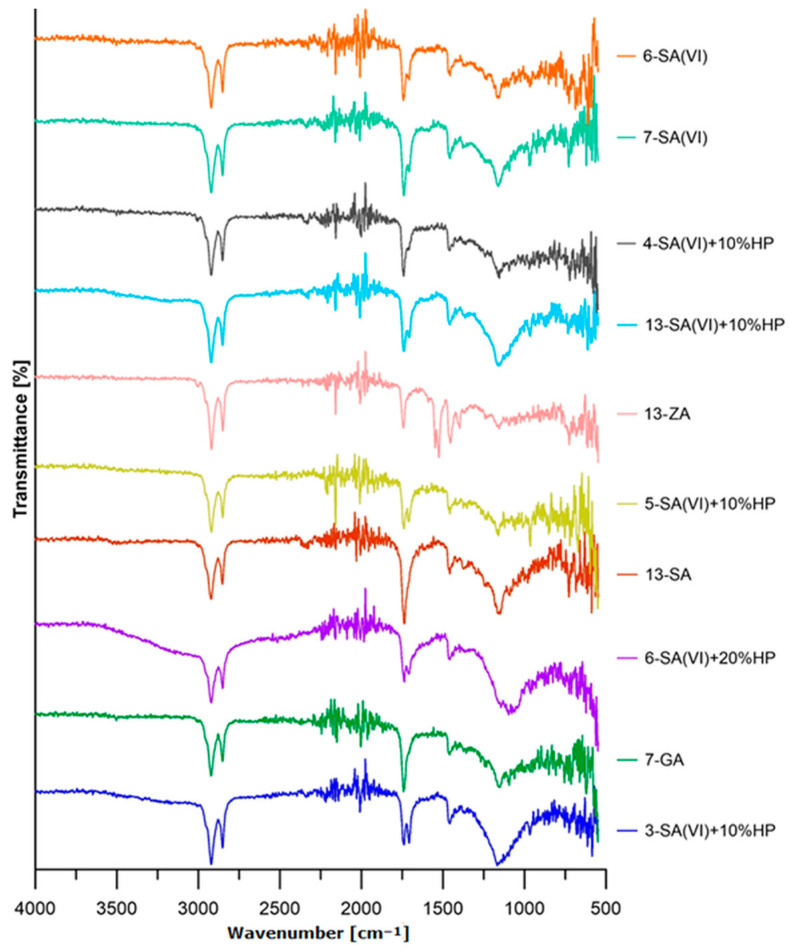
FT-IR spectra of selected binders.

Figure 3 shows the FT-IR spectra of selected binders. Each spectrum features bands at about 2920 cm^−1^ and 2850 cm^−1^, arising from C–H stretching vibrations in the methyl (-CH_3_) and methylene (-CH_2_-) groups present in long-chain hydrocarbons and in the triglycerides of waste frying oil [34,35]. The intensity of these bands is comparable across all samples analysed. The band near 1740 cm^−1^ corresponds to the C=O stretching vibration of carbonyl groups. In oils, this peak is characteristic of the ester functionality present in the triglycerides that make up the bulk of the oil [34,36]. The binder spectrum containing zinc acetate displays the weakest intensity of this peak, a factor that corresponded to the poor mechanical properties of the composites derived from it; conversely, that binder shows a more pronounced band at approximately 1458 cm^−1^, attributable to the deformation vibrations of −CH_2_− and −CH_3_ groups [34,37]. In the region around 1160 cm^−1^, bands assigned to C–O stretching in ester groups are observed, confirming the presence of these moieties [34,37,38]. A markedly lower intensity of these bands is observed in binders 14-ZA and 6-SA(VI) + 10% HP, which can be attributed to the curing temperature. The highest intensity, by contrast, occurs in sample 3-SA(VI) + 10% HP, cured for 12 h at 240 °C with a cat./WCOcat ratio of 0.15.

Comparing samples 7-SA(VI) and 7-GA—both cured at 240 °C for 18 h with cat./WCOcat = 0.15—shows that sulfuric(VI) acid fosters esterification more strongly than glycolic acid.

Comparing samples 5-SA(VI) + 10% HP and 5-SA(VI) + 20% HP—prepared under identical processing conditions but differing in hydrogen-peroxide content—reveals that the higher band intensities in 5-SA(VI) + 20% HP stem from the greater peroxide concentration. In sample 6-SA(VI), where sulfuric(VI) acid was the sole catalyst, additional S=O stretching bands appear in the 1100–1300 cm^−1^ region [39].

### 3.3. Mechanical Testing of the Oil Composites

Table 3 lists the compressive-strength values of the composite materials. These values vary considerably, ranging from 0.74 MPa for sample 13-ZA to 5.70 MPa for sample 13-SA(VI) + 20% HP. Both composites were produced by curing at 210 °C for 15 h with a cat./WCOcat ratio of 0.15, but they differed in the catalyst used.

The greatest number of composites were produced in processes 6 and 7, which were fired at 240 °C for 18 h. Their compressive strength did not reach the maximum recorded values, suggesting that intense sintering may have occurred, thereby lowering the compressive performance.

Raising the curing temperature to 240 °C is advantageous when pyrolytic oil is used as the binder base: in those systems, specimens cured at the higher temperature attained significantly higher compressive-strength values and exhibited smaller compressive deformations [40]. Pyrolytic oil consists chiefly of aromatic hydrocarbons such as benzene, toluene, and xylenes, along with polycyclic aromatic hydrocarbons (e.g., naphthalene) and an aliphatic-hydrocarbon fraction. This profile points to a mixture dominated by saturated and unsaturated aliphatic, aromatic, and cyclic compounds containing roughly 7–20 carbon atoms. The oil may also include hydrocarbon derivatives bearing heteroatoms—principally oxygen, sulphur, or nitrogen [41]. Compressive strength is markedly influenced by the choice of catalyst. Using sulfuric(VI) acid enabled the formation of a solid material at relatively low temperature and short curing time, as confirmed by statistical analysis. At the same time, the effect of adding hydrogen peroxide proved ambiguous: some samples catalysed with sulfuric(VI) acidplus hydrogen peroxide achieved higher compressive strength than those containing sulfuric acid alone (e.g., samples from processes 6, 7, 13), whereas in other cases the opposite trend was observed (e.g., samples from processes 5 and 11).

**Figure 4:**
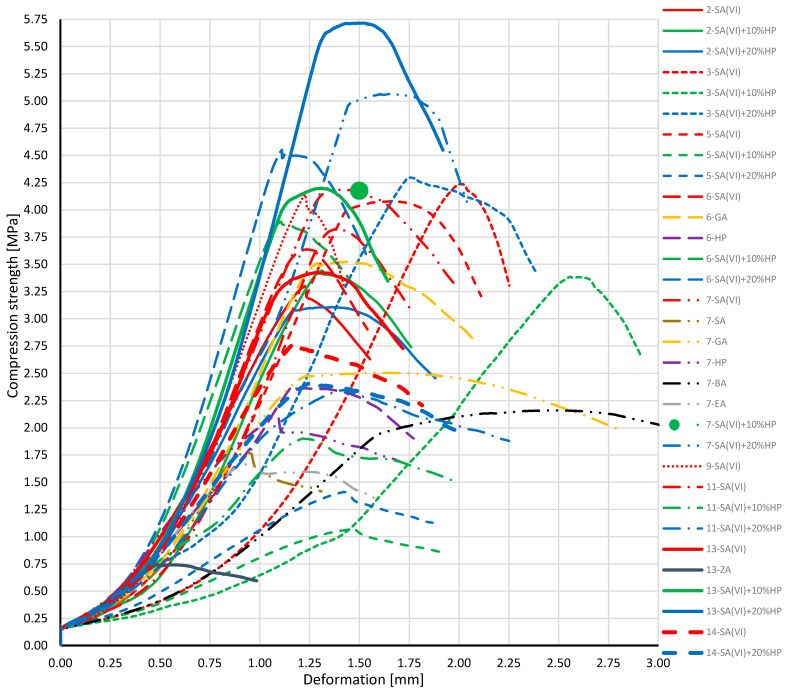
Compressive stress–strain curve for the composite specimen.

Figure 4 plots compressive strength versus specimen deformation. Owing to a random measurement failure, the strain reading for sample 7-SA(VI) + 10% HP was lost; consequently, only the compressive-strength value is shown for that sample.

When assessing the extent of solidification and the mechanical strength of the samples, the strain recorded during testing must also be taken into account. A high strain value can signal either flexibility or brittleness, depending on how the material deforms under load. Ideally, an oil-based composite should combine high compressive strength with low deformation. The lowest compressive strength—0.74 MPa—was obtained for sample 13-ZA, produced under the same processing parameters as the specimen that achieved the highest strength. This result highlights the critical role of catalyst choice: zinc acetate did not fulfil its intended function. One likely reason is that, as the salt of the weak acetic acid, it is insufficiently reactive to bring about full modification of the WCO. Moreover, zinc acetate may slow the kinetics of the key chemical reactions, weakening the developing network. Composites formulated with this catalyst also tend to exhibit higher porosity, which undermines structural integrity and further lowers compressive strength [42,43]. Similar conclusions can be drawn for hydrobromic acid, ethyl alcohol, and salicylic acid.

The lowest compressive-strength values were recorded for samples 13-ZA (210 °C, 15 h, cat./WCOcat = 0.15), 5-SA(VI) + 10% HP (180 °C, 18 h, cat./WCOcat = 0.15), and 5-SA(VI) + 20% HP (180 °C, 18 h, cat./WCOcat = 0.15). Conversely, relatively high strengths were obtained for specimens catalysed with sulfuric(VI) acid. This can be attributed to the strong oxidising power of sulfuric(VI) acid, which reacts vigorously with the WCO; moreover, its pronounced dehydrating action densifies the material, increasing hardness and, consequently, mechanical strength [42,43]. Compressive-strength tests on WCO-based composites enriched with antimicrobial additives showed that functional modifiers can, in some cases, influence mechanical performance. Composites whose WCO was catalysed with sulfuric(VI) acid (cat./WCOcat = 0.14, 210 °C, 18 h) and then doped with 4% thymol or salicylic acid achieved compressive strengths of 3.25 MPa and 2.37 MPa, respectively. By contrast, unmodified composites prepared under comparable conditions (cat./WCOcat = 0.10, 210 °C, 18 h) exceeded 4 MPa in compressive strength [22].

The bending strength of the specimens likewise varied, from 0.37 MPa for sample 11-SA(VI) + 10% HP (240 °C, 15 h, cat./WCOcat = 0.10) to 1.34 MPa for sample 7-SA(VI) + 20% HP (240 °C, 18 h, cat./WCOcat = 0.15) (Table 4). A low bending-strength value points to greater material brittleness, a higher susceptibility to cracking and deformation, and may result from the curing time—the longer the heat treatment, the lower the mechanical performance.

**Figure 5:**
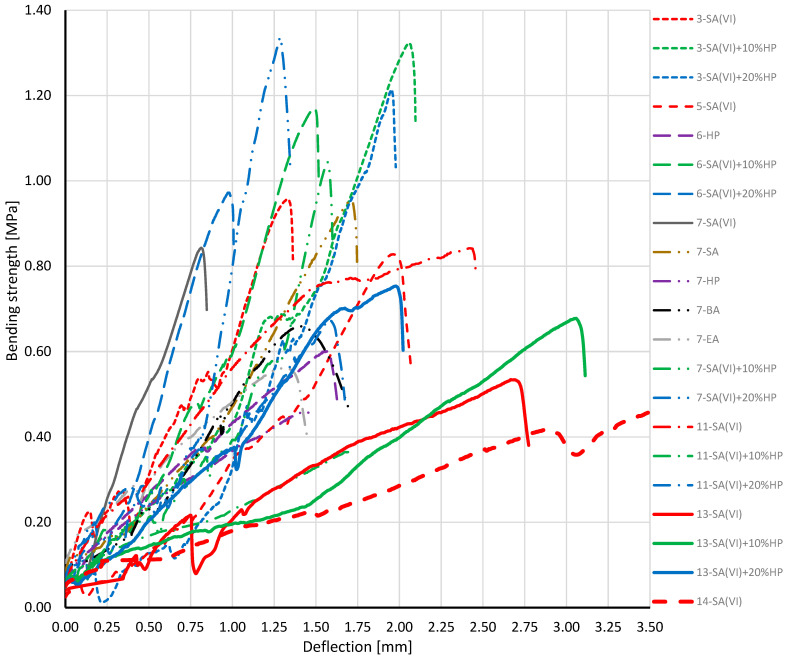
Bending-stress versus strain curve for the composite specimen.

Figure 5 displays the bending-strength results for specimens prepared with the various catalysts. The step-like drops visible on the plot signal the formation of micro-cracks within the specimen structure during the test.

The highest bending-stress values were obtained for samples from processes 3, 6 and 7, all of which contained sulfuric(VI) acid plus hydrogen peroxide. When composites produced under identical processing conditions (240 °C, 18 h, cat./WCOcat = 0.15) are compared, the most favourable strength is delivered by the formulation with sulfuric(VI) acid and 20% hydrogen peroxide. Sulfuric(VI) acid modifies the structure of the WCO, while the hydrogen peroxide—acting as a strong oxidiser—can, in synergy with sulfuric(VI) acid, oxidise constituents within the mixture, leading to the formation of more stable and mechanically robust structures.

### 3.4. Water-Absorption Test and Filtrate pH

The water-absorption test was carried out to evaluate the ability of the oil composites to take up water—a factor that directly affects their mechanical performance and long-term durability (Table 5). The pH of the de-ionised water used in the test was 5.6.

The pH of the solutions after incubation with the oil composites was similar across all catalyst types, ranging from 3.6 to 4.8. Water absorption, by contrast, varied markedly—from 3.0% to 8.9%. The lowest uptake (3.0%) was recorded for sample 6-GA, produced from WCO catalysed with glycolic acid. This result is consistent with glycolic acid’s dual role: in addition to catalysing (trans)esterification, the small bifunctional molecule (bearing both a hydroxyl –OH and a carboxyl –COOH group) can chemically integrate into the growing polymer network, thereby reducing porosity and limiting water ingress [44,45]. Consequently, the resulting composite develops a denser microstructure and is less permeable to water [46,47]. The highest water uptake was observed for composites 13-SA(VI) + 10% HP and 9-SA(VI), at 8.9% and 8.4%, respectively. Both specimens contained sulfuric(VI) acid as a strong mineral catalyst that remains outside the polymer network. Sulfuric(VI) acid can foster condensation or even degradation of oil components—e.g., by dehydrating and cleaving fatty-acid chains—which increases porosity and thus water absorption [48,49], leading to porosity, irregularities, or defects in the network structure of the resulting composites. High water absorption can diminish the mechanical performance of the materials under investigation [50,51]. Conversely, materials that do not absorb water tend to be more durable under harsh environmental conditions, and their mechanical properties can be maintained at the same level owing to the low water uptake.

### 3.5. Structural Investigation of the Solid Oil-Based Materials

**Figure 6:**
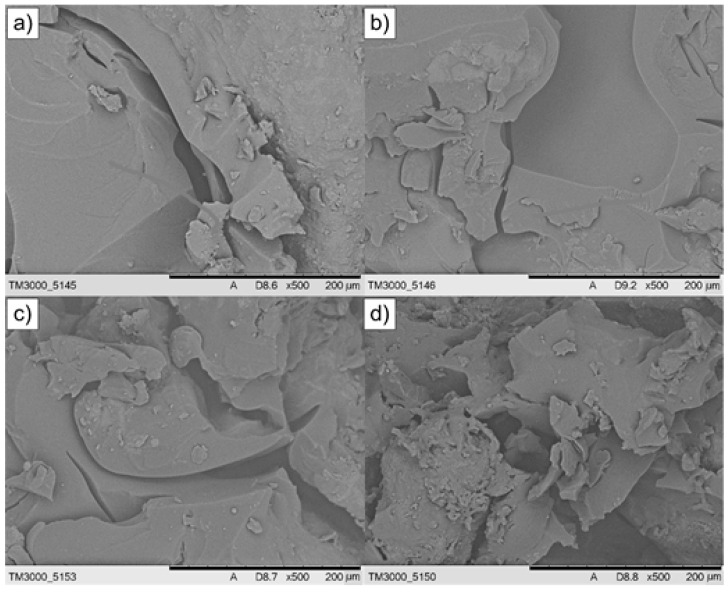
SEM micrographs of the composites: (**a**) 13-SA(VI) + 10% HP, (**b**) 6-GA, (**c**) 6-HP, (**d**) 6-SA(VI) + 20% HP.

Selected oil composites were examined by scanning electron microscopy. Figure 6 presents and compares the microstructures of composites prepared with sulfuric(VI) acid plus 10% hydrogen peroxide (210 °C, 15 h, cat./WCOcat = 0.15) and with glycolic acid (240 °C, 18 h, cat./WCOcat = 0.05) (Figure 6a,b). It also shows the structures of composites produced under identical processing conditions (240 °C, 18 h, cat./WCOcat = 0.05) using hydrogen peroxide alone (6-HP) and sulfuric(VI) acid with 20% hydrogen peroxide (6-SA(VI) + 20% HP).

The surfaces of specimens (a) and (b) exhibit a homogeneous, compact morphology composed of densely packed domains and only a few micro-cracks or sharp-edged features. Although the catalytic systems differ—a mixture of sulfuric(VI) acid with 10% hydrogen peroxide versus glycolic acid—both interact strongly with the WCO and accelerate solidification.

Sulfuric(VI) acid, as a strong inorganic acid, efficiently catalyses the esterification of free fatty acids [52,53,54], glycolic acid, by contrast, is a weak organic acid; it enters the esterification pathway by reacting with both free fatty acids and triglycerides [55].

Glycolic acid, being a weak organic acid, still participates in esterification by reacting with both free fatty acids and triglycerides. The composites shown in Figure 6c,d exhibit a large number of cracks. Although the 6-SA(VI) + 20% HP sample attains a higher compressive strength (4.55 MPa) than the 6-GA composite (3.52 MPa), its microstructure is less homogeneous. This finding suggests that hydrogen peroxide alone does not provide sufficient catalytic activity for WCO, which in turn affects the physicochemical properties of the resulting composite. In the presence of H_2_O_2_, unsaturated fatty acids are oxidised, leading to epoxidation of the C = C double bonds; introducing sulfuric(VI) acid into the reaction medium intensifies this process [56,57]. Moreover, thermal-degradation products of the oil—such as aldehydes and ketones—can themselves undergo further breakdown and oxidation [58].

### 3.6. Statistical Analysis of the Research Results

The datasets describing the physical properties of the composites were subjected to a classification procedure aimed at identifying major groups (clusters) of materials sharing similar characteristics. The clustering outcomes are presented as dendrograms. Ward’s method was applied to decide when two clusters were sufficiently similar to be merged.

**Figure 7:**
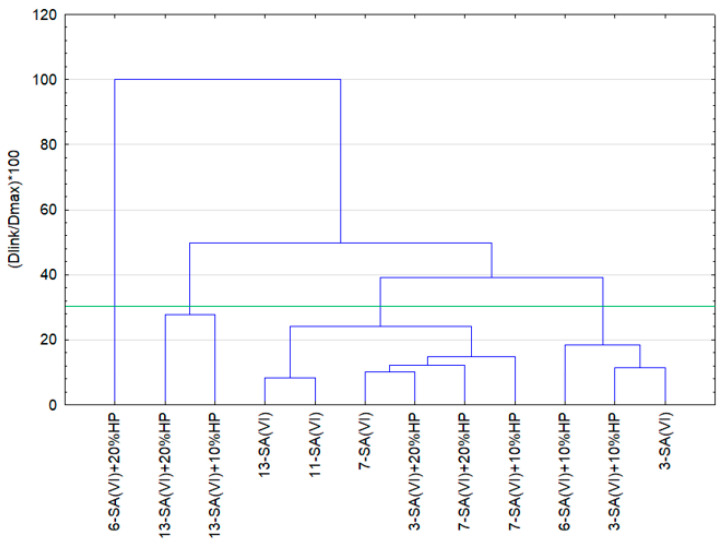
Agglomeration results for the oil composites.

**Figure 8:**
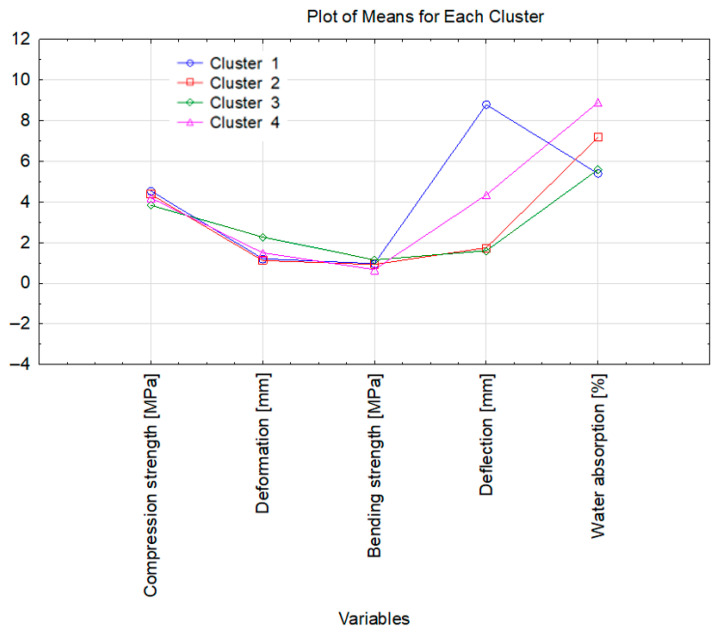
Mean values for the objects within each cluster.

Based on the agglomerative results, the specimens were classified into four clusters (Figure 7). These agglomeration findings then served as the starting point for a k-means analysis (Figure 8). The individual cases assigned to each cluster are listed in Table 6. It should be emphasised that the statistical calculations considered only those cases for which complete outcome data from all tests were available—that is, all dependent variables were known.

Materials containing sulfuric(VI) acid—either alone or in combination with hydrogen peroxide—exhibit comparable compressive strength. The greatest deformation (deflection) is seen in cluster 3, which may stem from the high curing temperature or the larger catalyst load used for those samples. Cluster 1 contains the composite with the single highest deflection; that specimen was produced with the minimum catalyst dosage (cat./WCOcat = 0.05). Lower deflection values occur in cluster 4, whose composites were cured at 210 °C, whereas clusters 2 and 3 comprise materials cured at 240 °C that show the smallest deflection. The plot also reveals distinct differences in water uptake among the clusters—from the lowest absorption in clusters 1 and 3 to the highest in cluster 4.

**Figure 9:**
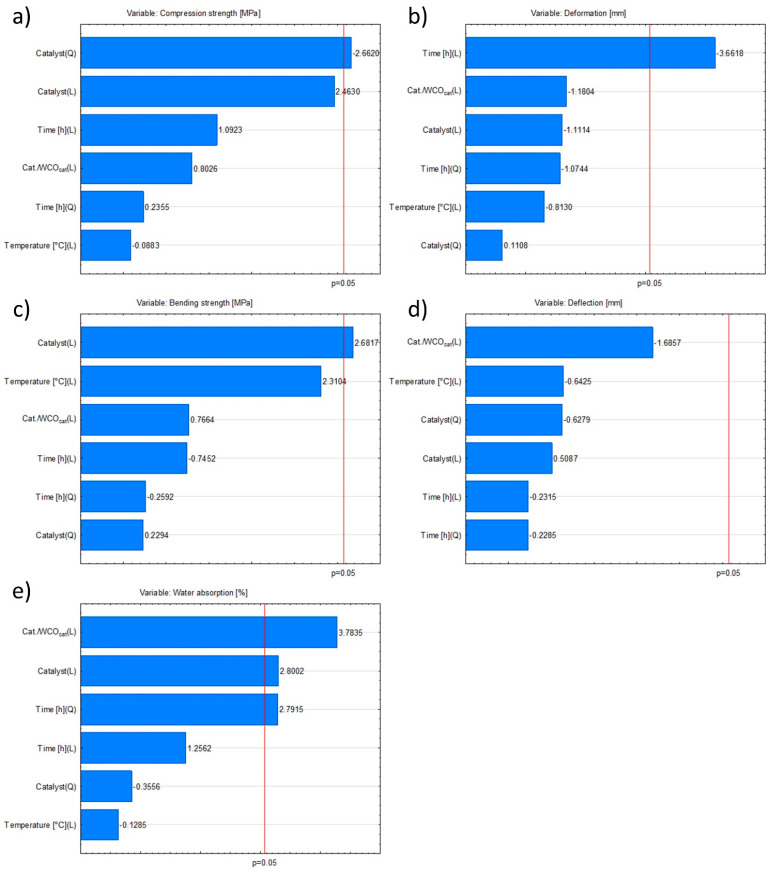
Pareto charts for the independent variables: (**a**) compressive strength, (**b**) deformation, (**c**) bending strength, (**d**) deflection, (**e**) water absorption.

On the basis of the Pareto charts it is possible to identify which of the investigated parameters (independent variables) or their interactions exert a statistically significant influence (α = 5%) on the dependent variables—namely the mechanical strength of the oil composites and their water absorption (Figure 9). For **compressive strength**, only the *quadratic* term of the variable *catalyst* proved significant, whereas for **bending strength** the significant factor was the *linear* term of the same variable. In the case of **deformation**, the sole significant contribution was the *linear* effect of curing time (Figure 9a–c).

None of the independent variables (curing time, curing temperature, catalyst type, or catalyst loading) had a significant effect on deflection. Water absorption, however, was governed by the linear effects of Cat./WCOcat and catalyst, together with the quadratic term of curing time (Figure 9d,e). Comparable trends were described by Ardila-Suárez and co-authors [59], who demonstrated the role of sulfuric(VI) acid as a catalyst in the formation of polymeric compounds. In their combinatorial experiment, they investigated the polymerisation of polyglycol, evaluating the effects of temperature and catalyst concentration. As with the composites derived from waste cooking oil, they demonstrated that carefully adjusting the process conditions allows the physicochemical properties of the resulting materials to be tailored.

**Figure 10:**
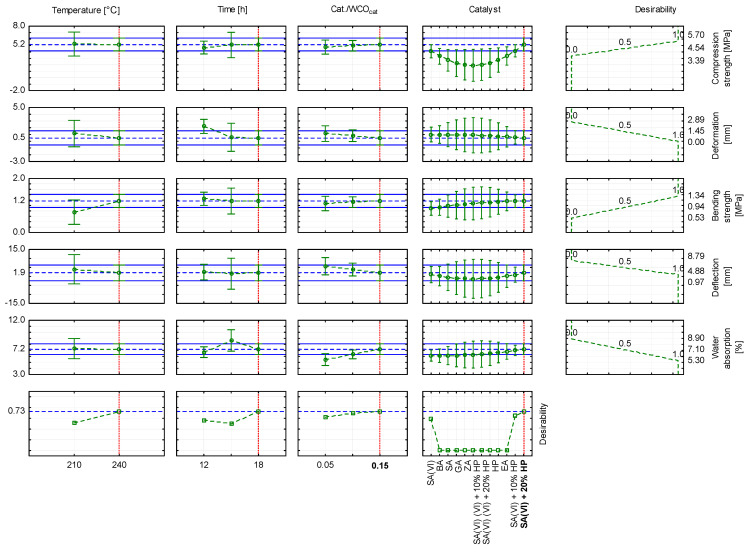
Approximation profile with the desirability function for producing oil composites using sulfuric(VI) acid + 20% H_2_O_2_.

**Figure 11:**
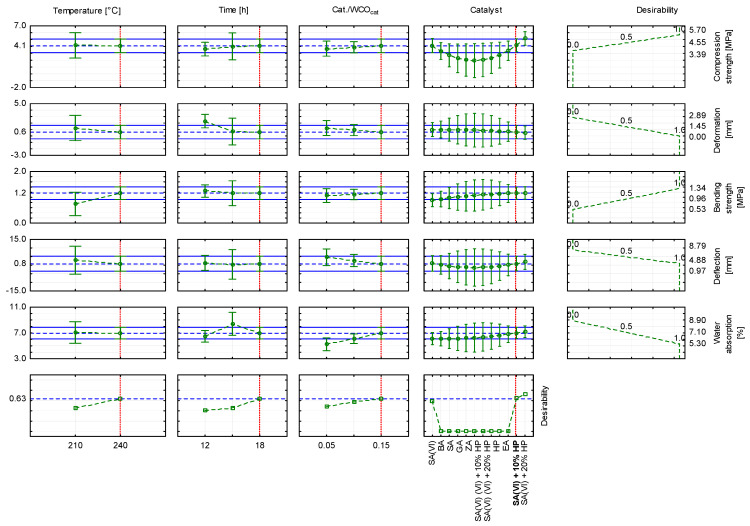
Approximation profile with the desirability function for producing oil composites using sulfuric(VI) acid + 10% H_2_O_2_.

**Figure 12:**
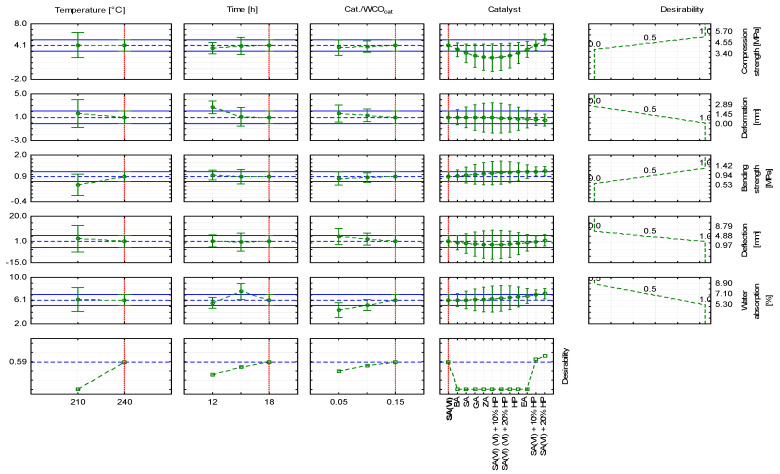
Approximation profile with the desirability function for producing oil composites using sulfuric(VI) acid.

To select the most favourable parameters for producing WCO-based oil composites, an approximation profile was generated from the model parameters and a desirability function was established (Figure 10 and Figure 11). The highest desirability value was obtained when a catalyst mixture containing sulfuric(VI) acid was used (Figure 12). When WCO served as the feedstock, the catalyst was a blend of sulfuric(VI) acid and 20% hydrogen peroxide, the Cat./WCOcat ratio was 0.15, the curing temperature was 240 °C and the dwell time was 18 h, the desirability reached 0.73 (Figure 10). The resulting composite specimen showed a compressive strength of 5.16 MPa with a deformation of 0.55 mm, a bending strength of 1.17 MPa with a deformation of 1.93 mm, and a water absorption of 7.20%. As the proportion of hydrogen peroxide in the catalytic mixture increased, desirability decreased. Using a catalyst composed of sulfuric(VI) acid and 10% hydrogen peroxide (Figure 11) lowered the desirability to 0.63; the composite obtained under these conditions displayed a compressive strength of 4.09 MPa with a deformation of 0.60 mm, a bending strength of 1.16 MPa with a deformation of 0.84 mm, and a water absorption of 6.98%. When sulfuric(VI) acid alone served as the catalyst (Figure 12), desirability fell to 0.59, and the resulting oil block exhibited a compressive strength of 4.11 MPa with a deformation of 1.01 mm, a bending strength of 0.89 MPa with a deformation of 0.98 mm, and a water absorption of 6.12%.

## 4. Conclusions

1. The present study demonstrated the feasibility of producing solid composites based on catalytically modified waste cooking oil (WCO) and sand. The proposed process offers a straightforward method of utilising WCO as a binder precursor, contributing to circular economy strategies and waste valorization.
2. Among the tested catalytic systems—including sulfuric(VI) acid, sulfuric(VI) acid/hydrogen peroxide blend, hydrobromic acid, glycolic acid, hydrogen peroxide, salicylic acid, ethyl alcohol, and zinc acetate—the formulations containing sulfuric(VI) acid exhibited the highest efficiency in binder formation and composite cohesion.
3. The composite formulated with sulfuric(VI) acid and 20 wt.% hydrogen peroxide and cured at 210 °C exhibited the highest compressive strength (5.70 MPa). The same formulation, when cured at 240 °C, achieved the greatest bending strength (1.34 MPa). These findings confirm the potential to tailor mechanical properties through catalyst selection and process parameters.
4. Although the developed materials exhibit lower mechanical strength than conventional construction materials, the values achieved are considered sufficient for selected non-structural applications. Potential uses include temporary construction elements, and fillers for modular systems.
5. The proposed composites offer environmental and economic benefits, including the reduction in reliance on virgin resources, mitigation of problematic waste streams, and suitability for low-infrastructure, decentralised production. These factors contribute to the material’s viability in sustainable building applications.
6. Certain challenges remain, notably the requirement for elevated curing temperatures and variability in WCO composition, which may affect process reproducibility and energy efficiency.
7. Future research should aim to improve binder reactivity at lower temperatures, investigate alternative or synergistic catalytic systems, and establish pre-treatment or standardisation protocols for WCO feedstock. Further studies on long-term durability, water resistance, and biodegradation under real environmental conditions are essential to validate the composites’ performance in practical applications.

## Figures and Tables

**Table 1:** Summary of the substances added to the process and their properties.

Designation	Substance	Physical State
SA(VI)	sulfuric(VI) acid	liquid solid (crystalline powder) solid
AA	acetic acid	liquid
SA	salicylic acid	solid (crystalline powder)
GA	glycolic acid	solid
ZA	zinc acetate	solid
HP	hydrogen peroxide	liquid
BA	hydrobromic acid	liquid
EA	ethyl alcohol	liquid
SA(VI) + 10% HP	sulfuric(VI) acid + 10% hydrogen peroxide	liquid
SA(VI) + 20% HP	sulfuric(VI) acid + 20% hydrogen peroxide	liquid
10% HP + SA(VI)	10% hydrogen peroxide + sulfuric(VI) acid	liquid
20% HP + SA(VI)	20% hydrogen peroxide + sulfuric(VI) acid	liquid
10% HP + AA	10% hydrogen peroxide + acetic acid	liquid
20% HP + AA	20% hydrogen peroxide + acetic acid	liquid

**Table 2:** Process parameters for preparing oil binders and (highlighted in grey) oil composites.

Process Number	Temperature [°C]	Time [h]	Cat./WCOcat
1	180	12	0.15
2	240	12	0.05
3	240	12	0.15
4	180	18	0.05
5	180	18	0.15
6	240	18	0.05
7	240	18	0.15
8	210	12	0.10
9	210	18	0.10
10	180	15	0.10
11	240	15	0.10
12	210	15	0.05
13	210	15	0.15
14	210	15	0.10

**Table 3:** Compressive strength of the oil composites.

Catalyst	Compressive Strength [MPa] for Samples from a Given Process								
	2	3	5	6	7	9	11	13	14
SA (VI)	3.30	4.24	4.08	3.77	4.19	4.13	3.83	3.42	2.76
SA (VI) + 10%HP	3.44	3.39	1.07	3.89	3.98		1.90	4.20	
SA (VI) + 20%HP	3.11	4.30	1.41	4.55	5.20		2.35	5.70	2.43
BA					2.16				
GA				3.52	2.50				
HP				2.38	2.11				
SA					1.77				
EA					1.67				
ZA								0.74	

**Table 4:** Bending strength of the oil composites.

Catalyst	Bending Strength [MPa] for Samples from a Given Process						
	3	5	6	7	11	13	14
SA (VI)	0.96	0.83		0.84	0.84	0.53	0.48
SA (VI) + 10%HP	1.32		1.17	1.04	0.37	0.68	
SA (VI) + 20%HP	1.18		0.97	1.34	0.67	0.75	
BA				0.66			
HP			0.60	0.46			
SA				0.95			
EA				0.57			

**Table 5:** Summary of water-absorption results and filtrate pH for the oil composites.

Sample	Process Parameters	Water Absorption [%]	pH
3-SA(VI)	240 °C, 12 h, Cat./WCOcat: 0.15	5.4	3.71
6-SA(VI)	240 °C, 18 h, Cat./WCOcat: 0.05	5.8	3.99
2-SA(VI)	240 °C, 12 h, Cat./WCOcat: 0.05	5.9	4.17
11-SA(VI)	240 °C, 15 h, Cat./WCOcat: 0.10	6.7	4.03
13-SA(VI)	210 °C, 15 h, Cat./WCOcat: 0.15	7.4	3.74
7-SA(VI)	240 °C, 18 h, Cat./WCOcat: 0.15	6.6	3.78
9-SA(VI)	210 °C, 18 h, Cat./WCOcat: 0.10	8.4	3.83
6-SA(VI) + 10%HP	240 °C, 18 h, Cat./WCOcat: 0.05	5.3	4.10
2-SA(VI) + 10%HP	240 °C, 12 h, Cat./WCOcat: 0.05	5.8	4.09
3-SA(VI) + 10%HP	240 °C, 12 h, Cat./WCOcat: 0.15	6.1	3.61
7-SA(VI) + 10%HP	240 °C, 18 h, Cat./WCOcat: 0.15	6.9	3.89
13-SA(VI) + 10%HP	210 °C, 15 h, Cat./WCOcat: 0.15	8.9	3.76
6-SA(VI) + 20%HP	240 °C, 18 h, Cat./WCOcat: 0.05	5.4	4.14
3-SA(VI) + 20%HP	240 °C, 12 h, Cat./WCOcat: 0.15	7.3	4.02
7-SA(VI) + 20%HP	240 °C, 18 h, Cat./WCOcat: 0.15	6.8	3.84
13-SA(VI) + 20%HP	210 °C, 15 h, Cat./WCOcat: 0.15	8.6	3.69
6-GA	240 °C, 18 h, Cat./WCOcat: 0.05	3.0	4.81

**Table 6:** Cluster members.

	**Cluster 1**	**Cluster 2**	**Cluster 3**	**Cluster 4**
Case	6-SA(VI) + 20%HP	7-SA(VI) + 10%HP	3-SA(VI)	13-SA(VI) + 10%HP
		7-SA(VI) + 20%HP	3-SA(VI) + 10%HP	
		3-SA(VI) + 20%HP	6-SA(VI) + 10%HP	
		7-SA(VI)		
		11-SA(VI)		
		13-SA(VI)		
		13-SA(VI) + 20%HP		

## Data Availability

The original contributions presented in this study are included in the article. Further inquiries can be directed to the corresponding author.

## Abbreviations

WCO	Waste Cooking Oil
SA(VI)	Sulfuric(VI) Acid
AA	Acetic Acid
SA	Salicylic Acid
GA	Glycolic Acid
ZA	Zinc Acetate
HP	Hydrogen Peroxide
BA	Hydrobromic Acid
EA	Ethyl Alcohol
FT-IR	Fourier-Transform Infrared Spectroscopy
SEM	Scanning Electron Microscopy
Cat./WCOcat	Catalyst-to-Waste-Cooking-Oil Mass Ratio
MPa	Megapascal
